# Institutional Needs

**Published:** 1913-05-03

**Authors:** 


### Institutional Needs.

A FOLDING CHART HOLDER.

An excellent folding chart holder which has recently been produced by The Scientific Press, Ltd., 28 and 29 Southampton Street, Strand, W.C., will be welcomed by maternity and district nurses. The new design will take a chart of standard size, and when not in use may be folded into four, the folds being kept in position by a band of elastic attached to the back. The advantage of it is that when opened out the holder is perfectly rigid, and the chart quite smooth, and that when folded the chart is kept clean, and it occupies but little space in the nurse's bag. It is the only "four-fold" chart holder on the market.

TUGMAN ELECTRICAL ANÆSTHETIC APPARATUS.

Mr. A. B. Tugman, dental surgeon, has designed a new apparatus, which can be obtained from the Dental Manufacturing Company, Ltd., Newman Street, London, W., for producing local anæsthesia for dental cases. The principle adopted is that of ionic medication. The electric current is applied by means of a compound headpiece, the extremities of which consist of sponge electrodes, which cover the region of the Gasserian ganglion; these electrodes are moistened with a solution of common salt before application. The conductor of the headpiece is connected with the negative pole of the battery, a dental syringe containing the anæsthetic fluid being connected with the positive. Mr. Tugman claims that this device makes it possible to produce analgesia with an anæsthetic solution of much lower percentage than ordinarily used, and, inasmuch as resistance is reduced to a minimum, the use only of a current of such low voltage as to preclude the possibility of irritation is required.

